# Response of Intestinal Microbiota to the Variation in Diets in Grass Carp (*Ctenopharyngodon idella*)

**DOI:** 10.3390/metabo12111115

**Published:** 2022-11-15

**Authors:** Gang Yang, Yuhan Xiang, Shanshan Wang, Yujie Tao, Lichen Xie, Lixin Bao, Kaikai Shen, Jiamin Li, Baoqing Hu, Chungen Wen, Vikas Kumar, Mo Peng

**Affiliations:** 1 Department of Fisheries Science, School of Life Science, Nanchang University, Nanchang 330031, China; 2 School of Animal Science and Technology, Jiangxi Agricultural University, Nanchang 330045, China; 3 Aquaculture Research Institute, Department of Animal, Veterinary and Food Sciences, University of Idaho, Moscow, ID 83844, USA

**Keywords:** *Ctenopharyngodon idella*, ecological network, homeostasis, microbial function

## Abstract

The intestinal microbiota is important for the nutrient metabolism of fish and is significantly influenced by the host’s diet. The effect of ryegrass and commercial diets on the intestinal microbiota of grass carp was compared in this study. In comparison to ryegrass, artificial feed significantly reduced the microbial diversity in the intestine, which was measured by a decrease in the observed OTUs, ACE, Shannon, and the InvSimpson index. Although grass carp fed with ryegrass and artificial feed shared a dominant phyla Firmicutes and Proteobacteria, the microbial composition was clearly distinguishable between the two groups. In grass carp fed with ryegrass, Alphaproteobacteria, Gammaproteobacteria, and Actinobacteria predominated, whereas Bacilli was significantly higher in the artificial feed group due to an increase in Weissella and an unassigned Bacillales bacteria, as well as a significant increase in a potential pathogen: *Aeromonas australiensis*. Grass carp fed with ryegrass exhibited a more complex ecological network performed by the intestinal bacterial community, which was dominated by cooperative interactions; this was also observed in grass carp fed with artificial feed. Despite this, the increase in *A. australiensis* increased the competitive interaction within this ecological network, which contributed to the vulnerable perturbation of the intestinal microbiota. The alteration of the microbial composition through diet can further affect microbial function. The intestinal microbial function in grass carp fed with ryegrass was rich in amino acids and exhibited an increased energy metabolism in order to compensate for a low-nutrient diet intake, while the artificial feed elevated the microbial lipid metabolism through the promotion of its synthesis in the primary and secondary bile acids, together with a notable enhancement of fatty acid biosynthesis. These results indicated that diet can affect the homeostasis of the intestinal microbiota by altering the microbial composition and the interspecific interactions, whilst microbial function can respond to a variation in diet.

## 1. Introduction

The intestine is the most important organ of digestion and absorption in fish, and it is also a complex ecosystem as it harbors an extremely diverse and complex microbial community [1,2,3]. It is widely acknowledged that the intestinal microbiota performs critical functions for the host, such as the production of digestion-related enzymes, vitamin synthesis, pathogen protection, and immune maturation [4,5,6]. The microbiota in the intestine provides a large number of fermented metabolites for the host, particularly herbivores [7,8,9]. The intestinal bacterial community is a complex micro-ecological system that is significantly influenced by several factors, including the nutritional components of the host’s food [6,10]. Since fishmeal is scarce, plant protein is frequently utilized in aquafeed; however, this may lead to intestinal inflammation and microbial dysbiosis in fish [11,12,13]. The number of species, their abundance, and their intricate microbial interactions play a critical role in the homeostasis of the intestinal microbiota [14]. The microbial interactions in the intestine are widely acknowledged to be dynamic in order to connect trillions of bacteria into a sophisticated ecological network [9,15,16]. Intestinal microbiota includes more than 100 times as many genes as the host, enabling it to encode a diverse range of enzymes with a variety of different metabolic capabilities [17]. Numerous studies have shown that the intestinal microbiota can act as an additional metabolic organ, contributing significantly to the host’s amino acid, glucose, energy, and lipid metabolism by producing fermentation by-products [18,19,20,21].

The herbivorous grass carp (*Ctenopharyngodon idella*) is China’s most productive freshwater fish. Numerous studies have characterized the grass carp’s intestinal microbiota [22,23,24,25] and cellulase-producing bacteria, which assists in the digestion of fish that are fed a high-fiber diet [7,8,26]. However, the existing knowledge about the bacterial community in the intestine is about the composition and function of microbial communities in grass carp; however, little is known about species-species interactions within the bacterial community. Our previous research looked at the microbiota in different parts of the intestine of grass carp, and we discovered that intricate interspecific interactions could boost the efficiency of the bacterial community fermentation [16]. It is widely acknowledged that the overuse of plant protein can disturb the interspecific interactions and result in a disorder of the intestinal bacterial community in fish. 

Diets not only provide nutrients to fish and the intestinal microbiota, but they also influence fish health by modulating the intestinal microbiome. The objective of this study was to investigate the response of grass carp’s intestinal microbiota to ryegrass and a commercial diet that is high in plant protein by evaluating the variation in composition, interspecific interactions, and metabolic functions of the intestinal bacterial community. Thus, the findings of this study may provide an in-depth understanding of the alteration of intestinal microbiota in grass carp in response to dietary variations, providing a theoretical basis for intervening in the intestinal microbiota to maintain grass carp health.

## 2. Materials and Methods

### 2.1. Sample Collection

The grass carp were raised in artificial ponds (area, 200 m^2^; depth mean, 1.5 m) in the Institute of Special Aquaculture, Yichun, China. The grass carp, in two ponds, were fed with ryegrass (GF group: 12.73% protein, 1.38% lipid, and 26.52% fiber of total dry matter) and a commercial diet, provided by Tongwei Co., Ltd., Chendu, China (CF group: 30% protein (mainly plant protein from soybean meal, cottonseed meal, and rapeseed meal), 5% lipid, and 10.13% fiber). The fish were fed to apparent satiation twice a day (8:30 and 16:00) for one month without antibiotics. Grass carp fed with ryegrass (*n* = 8, weight mean, 316.45 ± 32.62 g) and a commercial diet (*n* = 8, weight mean, 357.81 ± 68.31 g) were collected after 3 h of feeding in the morning. In each group, twelve fish in one pond were chosen randomly from among more than 20 caught fish, and the other fish were returned. Before dissection, the skin surface of the grass carp was sterilized with 70% ethanol to reduce contamination, and then the digesta from the middle intestine was collected and stored in sterile freezing tubes under −80 °C. The experimental protocols of grass carp handling and sampling have been approved by the department of Laboratory Animal Science, Nanchang University (Approval Number: NCU-208-2021). 

### 2.2. Illumina Sequencing of Bacterial 16S rRNA Gene

PowerFecal™ DNA Isolation Kit (MoBio Laboratories, Inc., Carlsbad, CA, USA) was used for DNA extraction of digesta samples. Amplification of the 16S rRNA V3-V4 region was performed as described previously with barcoded fusion primers of 341F and 805R [27]. High-throughput sequencing was performed using the Illumina HiSeq platform at Novogen Co., Ltd., Beijing, China. All the sequencing data can be found in the Sequence Read Archive (SRA) database at NCBI under accession number PRJNA880788.

### 2.3. Bioinformatics and Statistical Analysis

The raw sequences were sorted into different samples according to the barcodes by using the BIPES pipeline, followed by a quality-control step to remove any low-quality amplicon sequences by VSEARCH [28]. The clean sequences were then clustered into operational taxonomic units (OTUs) with a 99% sequence similarity and annotated using the Ribosomal Database (rdp_16s_v16_sp). A total of 1,369,277 effective sequences and 1201 OTUs were generated from all samples. Alpha diversity and the relative abundance of taxa analyses were calculated by R software v 4.1.3. The Wilcoxon test was used to test the α-diversity index, and the relative abundance of taxa using R software. Treemap was used to visualize the significantly abundant OTU’s, the annotated taxonomy, the P-value, and the relative abundance, in which the size of the bubbles indicated the relative abundance of the raw read counts [29]. Principal coordinates analysis (PCoA), based on the Bray–Curtis dissimilarity analyses, were employed to visualize the bacterial community structure and the differences in the bacterial community was calculated by Permutational analysis of variance (PERMANOVA) based on the Bray–Curtis distance [30]. 

Using abundance profiles of the individual OTUs, a molecular ecological network analysis was performed to evaluate bacterial species-to-species interactions within a community (http://ieg2.ou.edu/MENA (accessed on 15 July 2022)). A Random Matrix Theory (RMT)-based approach was used for an ecological network construction and topological role identification [31]. The network was visualized using Circos and Cytoscape 3.9.0. Based on a modularity property, each network was separated into modules by the fast greedy modularity optimization. According to values of within-module connectivity (Zi) and among module connectivity (Pi), the topological roles of different nodes can be categorized into four types: peripherals (Zi ≤ 2.5, Pi ≤ 0.62), connectors (Zi ≤ 2.5, Pi > 0.62), module hubs (Zi > 2.5, Pi ≤ 0.62) and network hubs (Zi > 2.5, Pi > 0.62).

Functional gene and Kyoto Encyclopedia of Genes and Genomes (KEGG) pathways were predicted using PICRUSt2 software [32] against a Greengenes reference database (Greengenes 13.5). The non-metric multidimensional scaling (NMDS) and analysis of similarity (ANOSIM) were used to evaluate the overall differences in predicted bacterial functional compositions based on the Bray–Curtis distance at KEGG orthology (KO) level [33]. A two-sided Welch’s *t* test was used to identify significant different metabolic pathways in the two groups by software STAMP, with *p* < 0.05 considered significant. 

## 3. Results

### 3.1. Diversity and Composition of the Bacterial Community

**Figure 1:**
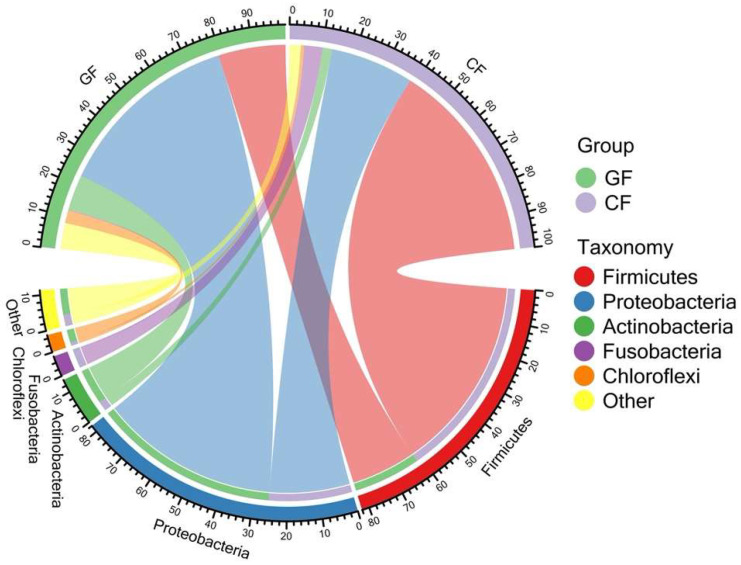
Chord diagram exhibited the relative abundance of bacterial phyla above ≥ a cutoff value of 2%.

**Figure 2:**
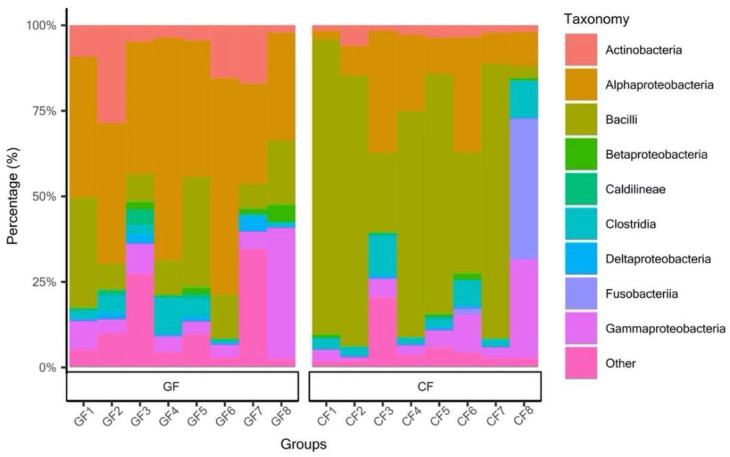
Relative abundances of the top 10 bacterial classes.

The bacterial community of grass carp in the GF group displayed a significantly higher value in the number of observed OTUs, ACE, Shannon, and InvSimpson index when compared to grass carp fed with the commercial diet (*p* < 0.05), whereas no significant differences were observed in chao1 (Table 1). The most observed phylum of the bacterial communities included Firmicutes (GF: 20.49%; CF: 61.59%), Proteobacteria (GF: 56.59%; CF: 25.42%), Actinobacteria (GF: 10.77%; CF: 2.97%), Fusobacteria (GF: 0.24%; CF: 5.62%), and Chloroflexi (GF: 3.93%; CF: 1.02%) were detected in the mid-intestine (Figure 1 and Table S1). Specifically, Alphaproteobacteria (43.84%), Bacilli (16.11%), Actinobacteria (10.77%), and Gammaproteobacteria (9.51%) took dominance in the middle intestine of grass carp fed with ryegrass, while the grass carp in the CF group were enriched with classes of Bacilli (55.58%), Alphaproteobacteria (16.63%), Gammaproteobacteria (7.51%), Fusobacteria (5.62%), and Clostridia (5.24%) in the intestine (Figure 2 and Table S2). 

**Figure 3:**
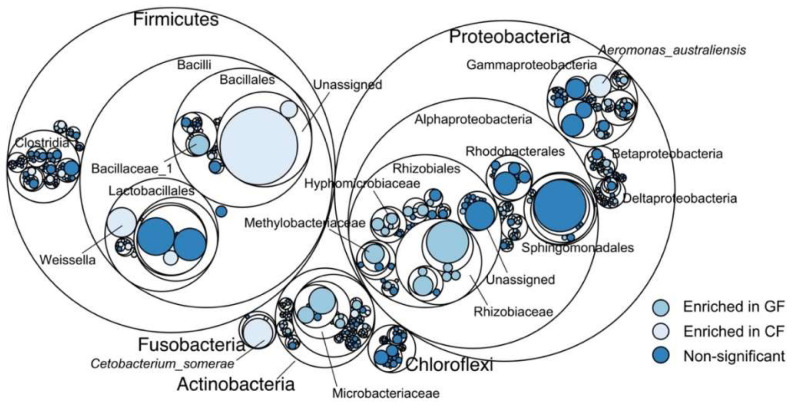
Maptree plot descriptions of the taxonomic differences based on 16S rRNA sequences. The largest circles represent phyla level, the inner circles represent class, order, family, and genus for panel.

**Figure 4:**
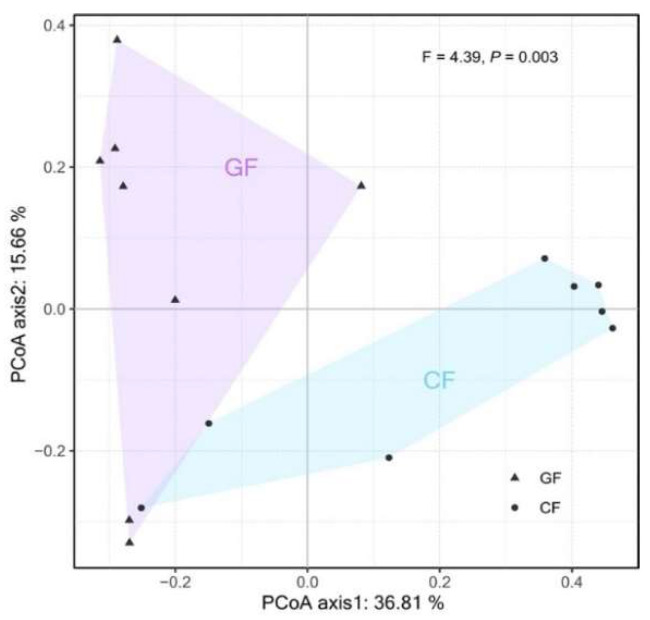
Principal coordinates analysis (PCoA) plot based on Bray–Curtis dissimilarity visualizing dissimilarities in the intestinal bacterial community of grass carp fed with ryegrass and a commercial diet.

As shown in Figure 3, grass carp in the GF group were significantly rich in Rhizobiales (mainly Rhizobiaceae, Rhizobiaceae, and Methylobacteriaceae), Microbacteriaceae from Actinobacteria, and Bacillaceae_1 from Bacilli (*p* < 0.05), whereas Weissella from Lactobacillales (Bacilli), *Cetobacterium_somerae* from Fusobacteriia, *Aeromonas_australiensis* from Gammaproteobacteria, and unassigned bacteria from Bacillales (Bacilli) dominated the bacterial community of grass carp from the CF group (*p* < 0.05). Additionally, the PCoA analysis exhibited a clear separation in the bacterial communities between the GF and the CF groups at the OTU level, and a significant difference was further revealed using PERMANOVA (*p* = 0.003, Figure 4). 

### 3.2. Ecological Network Analysis

**Figure 5:**
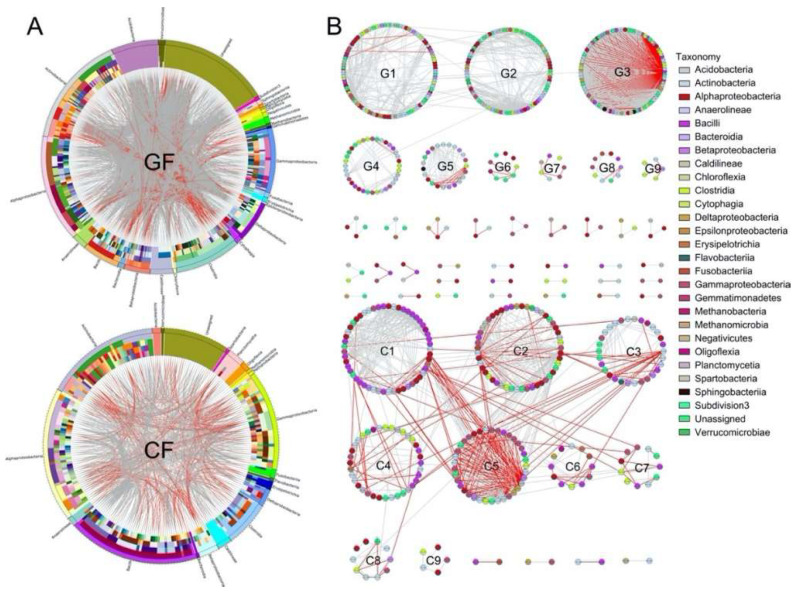
Circular plot (**A**) and ecological network (**B**) descriptions of the interaction between species within intestinal bacterial community. The width of the bars represents the abundance of each taxon. The bands with different colors demonstrate the source of different genera. The taxonomic levels were class, order, family, genera, and species from the outside to the inside of the circle. Each node in the network graph indicates one OTU. Colors of the nodes indicate different major classes. The edges (gray edge = positive interaction and red edge = negative interaction) inside the circle and ecological network represent the interactions between species.

A circos plot displayed a classified composition and species-species interactions within the bacterial community (Figure 5A), which consisted of different OTUs from 27 bacterial classes (Table 2). The GF network represented 441 OTUs and 2946 edges (gray edges: 2099; red edges: 847), and the CF network displayed 252 OTUs and 856 edges (gray edges: 592; red edges: 264). The gray and red edges indicated the positive and negative interactions between two OTUs. The GF network recorded major OTUs (≥20) from Actinobacteria (69 OTUs), Alphaproteobacteria (66 OTUs), Clostridia (41 OTUs), Gammaproteobacteria (40 OTUs), Acidobacteria (29 OTUs), Deltaproteobacteria (25 OTUs), and Bacilli (23 OTUs), whereas the major OTUs from Alphaproteobacteria (47 OTUs), Bacilli (46 OTUs), Actinobacteria (40 OTUs), and Gammaproteobacteria (35 OTUs) were observed in the CF network. 

**Figure 6:**
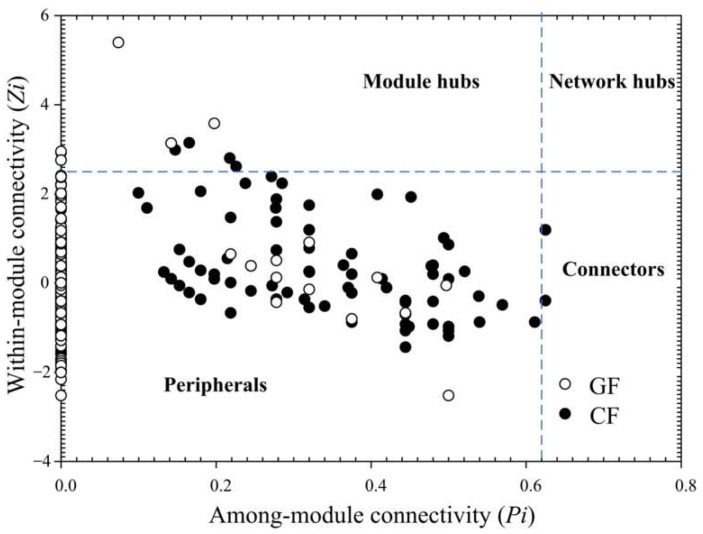
*Z-P* plot showing the distribution of OTUs based on their topological roles.

In the ecological network, an RMT-based approach was employed to delineate separate modules. Strikingly, in Figure 5B, the ecological network within the bacterial community of the grass carp fed with ryegrass consisted of 9 modules (≥5 nodes), and 4 larger sub-modules with ≥ 30 nodes were G1 (105 OTUs), G2 (86 OTUs), G3 (87 OTUs), and G4 (36 OTUs). Similarly, the CF network also had 9 modules with more than 5 nodes, and 5 sub-modules with ≥ 30 nodes were observed including C1 (53 OTUs), C2 (50 OTUs), C3 (30 OTUs), C4 (36 OTUs), and C5 (37 OTUs). Negative interactions were observed dominantly within the G3 and C5 sub-modules, whilst many negative edges were recorded between C1 and C5, or C3 and C5 sub-modules. Each specie performed different topological roles in the ecological network, in which most of the nodes were peripherals and several nodes performed as module hubs or connectors (Figure 6). As shown in Table 3, the GF network recorded 7 module hubs in G1 (3 OTUs), G2 (2 OTUs) and G4 (2 OTUs) sub-modules, among which these nodes were from Alphaproteobacteria (OTU_891, OTU_901 and OTU_911), Actinobacteria (OTU_568), Chloroflexi (OTU_756), Erysipelotrichia (OTU_376), and Gammaproteobacteria (OTU_153). In CF network, 4 module hubs (OTU_149, OTU_549, OTU_892 and OTU_1171) and 3 connectors (OTU_376, OTU_522 and OTU_894) were observed in C2 (3 OTUs), C3 (1 OTU) and C4 (3 OTUs) sub-modules, among which these nodes were from Alphaproteobacteria (OTU_892 and OTU_894), Actinobacteria (OTU_549), Clostridia (OTU_1171), Erysipelotrichia (OTU_376), Gammaproteobacteria (OTU_149), and Planctomycetia (OTU_522).

### 3.3. Functional Predictions of Intestinal Microbiota with PICRUSt2

**Figure 7:**
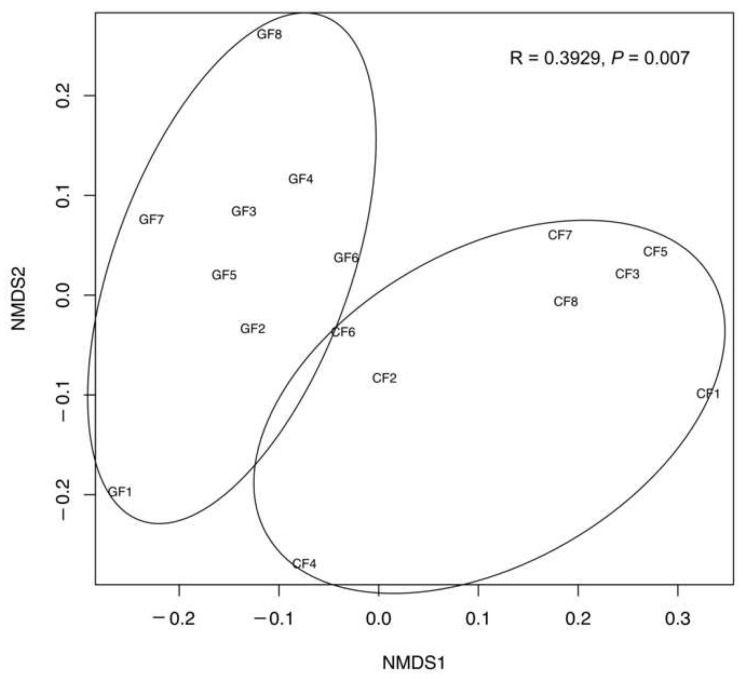
Non-metric multidimensional scaling (NMDS) plot visualizing bacterial functional community dissimilarities using Bray–Curtis distance.

**Figure 8:**
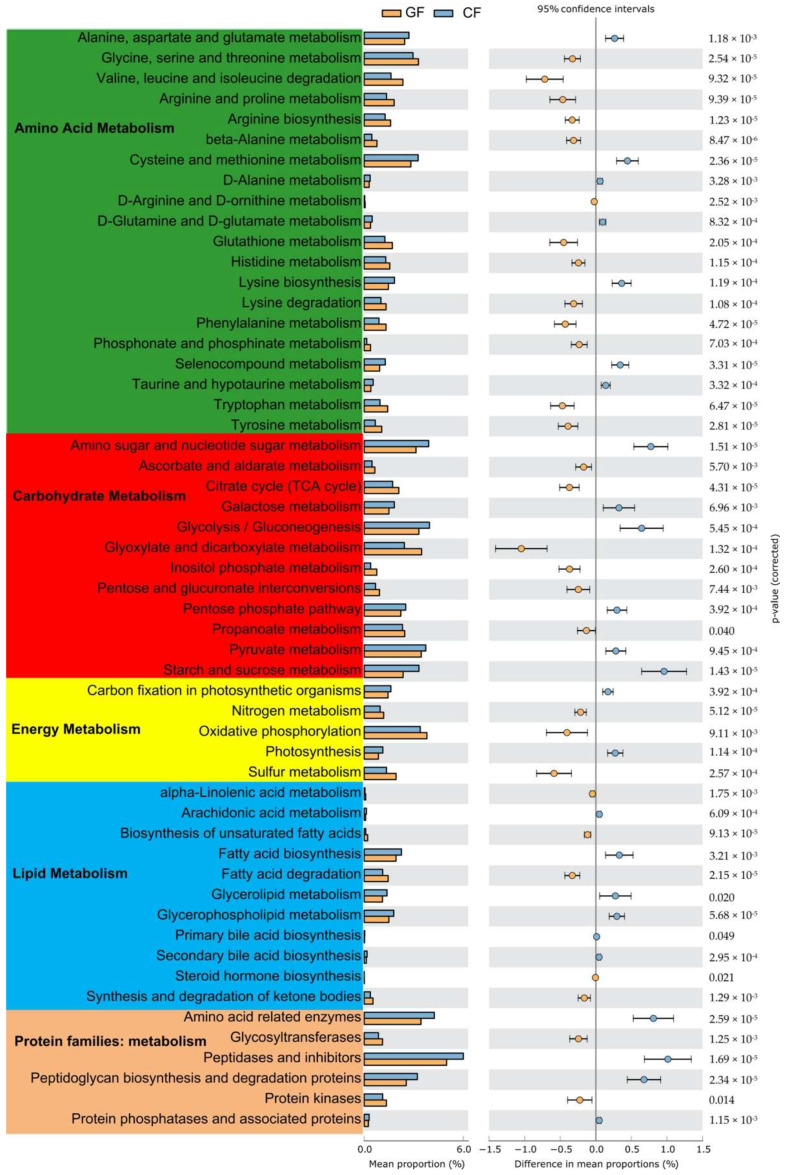
Significant changes in intestinal bacterial Kyoto Encyclopedia of Genes and Genomes (KEGG) pathways between ryegrass diet-fed and commercial diet-fed grass carp using the response ratio method at a 95% confidence interval (CI).

The NMDS analysis exhibited a clear distinction in the functional composition of the intestinal microbiota between the GF and the CF groups at a KO level, and an analysis of similarity (ANOSIM) further confirmed the remarkable differences in the bacterial functional composition between the GF and the CF groups (*p* = 0.007, Figure 7). To study the microbial metabolic function, the KEGG functional categories were analyzed, including amino acid metabolism, carbohydrate metabolism, energy metabolism, lipid metabolism, and protein families metabolism (Figure 8). A significant difference in 47 pathways and 6 protein families, which are involved in nutrient metabolism, were observed between the GF and the CF groups (*p* < 0.05). In particular, the bacterial communities in the grass carp fed with ryegrass was significantly rich in 13 pathways in amino acid metabolism, 6 pathways in carbohydrate metabolism, 3 pathways in energy metabolism, 5 pathways in lipid metabolism, and 2 protein families related to metabolism (*p* < 0.05). The dietary commercial diet notably promoted bacterial metabolic function including 7 pathways in amino acid metabolism, 6 pathways in carbohydrate metabolism, 2 pathways in energy metabolism, 6 pathways in lipid metabolism, and 4 protein families related to metabolism in grass carp (*p* < 0.05). 

## 4. Discussion

The importance of intestinal microbiota in host health has been highlighted in recent decades, owing to its benefits to nutrient metabolism and immune maturation [4,34,35]. It is widely accepted that diets can influence the composition of the intestinal bacterial community because microbes require nutrients and energy from the food consumed by hosts [36]. Grass carp feed on aquatic weeds in the wild, and previous research found that grass carp fed a Sudan grass diet were rich in Firmicutes, Proteobacteria, Fusobacteria, and Actinobacteria in the intestine [37]. In the current study, Proteobacteria, Firmicutes, and Actinobacteria were found to be the most prevalent phyla in the intestines of grass carp fed with ryegrass. Grass carp, the most important aquaculture species in China, are primarily fed an artificial diet rich in plant protein. Previous research has shown that commercially formulated feed can significantly alter the structure of the intestinal bacterial community in grass carp, resulting in a decrease in microbial biodiversity [37]. Indeed, the current findings show that the artificial feed reduced diversity, and the proportion of Alphaproteobacteria and Actinobacteria, while increasing Bacilli. Furthermore, in this study, grass carp fed a high plant protein diet had a significantly higher proportion of *Aeromonas australiensis* from Gammaproteobacteria. *Aeromonas* are Gram-negative microbes that live in aquatic environments and have been identified as an opportunistic pathogen for fish [38,39]. Previous research has suggested that *Aeromonas* sp. infection can cause intestinal inflammation and mucosal barrier function damage in grass carp [40,41], crucian carp [42], and Nile Tilapia (*Oreochromis niloticus*) [43]. As a result, the significant increase in *A. australiensis,* caused by the artificial feed, increased the potential threat to the health of grass carp in this study. 

As species perform similar or complementary functions, competitive and cooperative interactions occur between different species in the same habitat, for example competition to occupy more space and to obtain more nutrients [44]. The intestinal bacterial community can form a complex ecological network based on these interspecific interactions, allowing the intestinal microbiota to maintain a dynamic homeostasis in the host [14]. The microbial interactions were clearly degraded in the grass carp fed with an artificial diet due to a decrease in the microbial diversity, which can have a negative effect on the stability of a bacterial community because the intestinal microbiota is more easily disturbed by external factors. A cooperation dominated community is thought to be more stable because cooperative interactions are more resistant to population perturbations in spatial conditions, whereas competitive interactions are more vulnerable to disruption [45,46]. Indeed, the current findings suggested that cooperation dominated the intestinal bacterial community of grass carp, whereas the increased percentage of competitive interactions in the group fed with the artificial diet can be attributed to the significant increase in the opportunistic pathogen *A. australiensis*. Despite the fact that the grass carp, in two groups, showed distinctive submodules in the ecological networks, the networks in this study were primarily made up of the dominating microbial flora of each group. From an ecological perspective, connections, and module hubs generalists act as structural and functional keystones and play a crucial role in sustaining the ecological network [47]. According to the latest findings, the network’s generalist population was unaffected by the artificial diet. By weakening interspecific relationships and reducing cooperation in the ecological network, the artificial diet may collectively have a detrimental influence on the gut microbiota homeostasis. 

Since the genes of microbes encode several enzymes involved in protein, carbohydrate, lipid, and energy metabolism, microbial fermentation plays a critical role in the host’s nutritional metabolism [35,48,49]. The structure and operation of the gut bacterial community can adjust to changes in the dietary environment in a similar manner [36]. Due to differences in gene expression across the different microbes in the current study, dietary ryegrass, and artificial feed, as expected, shaped two distinctly different intestinal microbiota structures in grass carp. These differences were accompanied by a notable variation in the metabolic activities of the microbial community. Fish were frequently affected with the issue of aberrant fat deposition during breeding due to the use of a high-protein and a high-fat artificial feed. [50,51]. Evidence in both humans and animals has shown a robust correlation between obesity and a high Firmicutes ratio [52,53]. Here, compared to a ryegrass diet, the artificial feed did increase the proportion of Firmicutes in grass carp. This was followed by an increase in lipid metabolism, specifically an improvement in the biosynthesis of fatty acids, glycerols, and glycerospholipids, as well as a promotion of the production of primary and secondary bile acids. However, when grass carp were fed with ryegrass to supplement their low-nutrient diet, the microbial function related to amino acid and energy metabolism was more active. Furthermore, it has been demonstrated that microbial fermentation of complex, non-digestible dietary carbohydrates aids the host in obtaining useable forms of energy from a plant-based diet [8]. Grass carp are generally considered herbivorous, but no gene in the grass carp genome encodes an enzyme to digest cellulose, which comprises the principal component of a plant-based diet [54]. Evidence has confirmed that the microbial function of metabolizing cellulose plays an important role in the nutrient metabolism of an herbivore, and the abundant enzymes involved in the digestion of complex carbohydrates were also observed in the intestinal microbiota of grass carp [8]. Here, grass carp in the ryegrass diet group displayed a higher proportion of Actinobacteria, which proved to harbor an increased level of carbohydrate enzyme genes for the degradation of cellulose [55]. Nevertheless, it was shown that grass carp fed with an artificial feed, where starch was the predominant source of carbohydrate, had an increased microbial starch and sucrose metabolism. Additionally, a group of microorganisms that are interdependent on the effectiveness of fermentation are necessary for the microbial process of fermentation [9,56]. Therefore, the increased cooperative interaction may encourage the microbial fermentation of a ryegrass diet, assisting the grass carp to obtain more nutrients and energy. However, the microbial function in grass carp fed with the artificial diet was more susceptible to diseases in this study because of the per durability in the bacterial population. 

The modification of intestinal microbiota is an effective method for keeping fish healthy because it plays a significant role in the metabolism of nutrients and the host’s health. In fact, several studies have shown that by managing the homeostasis of fish’s intestinal microbiota with functional feed additives improves fish health [57,58]. The current study evaluated the intestinal bacterial communities of grass carp fed with an artificial diet and ryegrass, and it would serve as a theoretical guide for the control of grass carp intestinal microbiota.

## 5. Conclusions

Intestinal bacterial community characteristics in grass carp were altered by dietary ryegrass and artificial feed. An opportunistic pathogen, *A. australiensis*, was found to drastically increase in the artificial feed diet. Additionally, artificial feed may impact the lipid metabolism of grass carp by raising the ratio of Firmicutes and decreasing the disturbance resistance of intestinal microbiota.

## Figures and Tables

**Table 1:** Alpha diversity estimates of the bacterial communities (mean ± S.E.; *n* = 8) ^1^.

Groups	Observed OTUs	Chao1	ACE	Shannon	InvSimpson
GF	518.88 ± 21.96 ^b^	618.72 ± 16.8	605.7 ± 17.79 ^b^	3.78 ± 0.23 ^b^	16.8 ± 3.49 ^b^
CF	304.13 ± 41.13 ^a^	351.08 ± 51.7	344.53 ± 50.91 ^a^	2.76 ± 0.2 ^a^	5.86 ± 1.35 ^a^

^1^ Values in the same row with the different superscript are significantly different (*p* < 0.05).

**Table 2:** The composition of the ecological network.

Index	GF	CF
Acidobacteria	29	3
Actinobacteria	69	40
Alphaproteobacteria	66	47
Anaerolineae	9	3
Bacilli	23	46
Bacteroidia	4	1
Betaproteobacteria	16	7
Caldilineae	14	5
Chloroflexia	2	0
Clostridia	41	16
Cytophagia	4	0
Deltaproteobacteria	25	6
Epsilonproteobacteria	1	0
Erysipelotrichia	3	3
Flavobacteriia	0	1
Fusobacteriia	3	3
Gammaproteobacteria	40	35
Gemmatimonadetes	1	0
Methanomicrobia	4	0
Methanobacteria	2	2
Negativicutes	3	
Oligoflexia	2	3
Planctomycetia	1	6
Spartobacteria	2	1
Sphingobacteriia	3	0
Subdivision3	2	0
Verrucomicrobiae	4	1
Unassigned	68	23
Total number of OTUs	441	252
The number of modules (≥5 OTUs)	9	9
The number of module hubs	7	4
The number of connectors	0	3
The number of gray edges	2099	592
The number of red edges	847	264
Total number of edges	2946	856

**Table 3:** Topological roles of intestinal microbiota in grass carp.

Treatment	Topological Roles	OTUs	Module Number	Phylogenetic Associations
GF	Module hubs	OTU_376	1	Erysipelotrichia
	Module hubs	OTU_568	4	Actinobacteria
	Module hubs	OTU_891	2	Alphaproteobacteria
	Module hubs	OTU_756	2	Chloroflexi
	Module hubs	OTU_153	4	Gammaproteobacteria
	Module hubs	OTU_901	1	Alphaproteobacteria
	Module hubs	OTU_911	1	Alphaproteobacteria
CF	Module hubs	OTU_549	3	Actinobacteria
	Module hubs	OTU_1171	2	Clostridia
	Module hubs	OTU_892	2	Alphaproteobacteria
	Module hubs	OTU_149	2	Gammaproteobacteria
	Connectors	OTU_522	4	Planctomycetia
	Connectors	OTU_894	4	Alphaproteobacteria
	Connectors	OTU_376	4	Erysipelotrichia

## Data Availability

The data presented in this study are available in the main article and the Supplementary Materials.

## Supplementary Materials

The following supporting information can be downloaded at: https://www.mdpi.com/article/10.3390/metabo12111115/s1, Table S1: Relative abundance of different bacterial phyla in grass carp, Table S2: Relative abundance of different bacterial classes in grass carp.

## References

[1] Tarnecki A.M., Burgos F.A., Ray C.L., Arias C.R. (2017). Fish intestinal microbiome: Diversity and symbiosis unravelled by metagenomics. J. Appl. Microbiol..

[2] Talwar C., Nagar S., Lal R., Negi R.K. (2018). Fish gut microbiome: Current approaches and future perspectives. Indian J. Microbiol..

[3] Kim P.S., Shin N.-R., Lee J.-B., Kim M.-S., Whon T.W., Hyun D.-W., Yun J.-H., Jung M.-J., Kim J.Y., Bae J.-W. (2021). Host habitat is the major determinant of the gut microbiome of fish. Microbiome.

[4] Costa M.C., Weese J.S. (2018). Understanding the intestinal microbiome in health and disease. Vet. Clin. N. Am. Equine Pract..

[5] Dupont H.L., Jiang Z.-D., Dupont A.W., Utay N.S. (2020). The intestinal microbiome in human health and disease. Trans. Am. Clin. Climatol. Assoc..

[6] Power S.E., O’Toole P.W., Stanton C., Ross R.P., Fitzgerald G.F. (2014). Intestinal microbiota, diet and health. Br. J. Nutr..

[7] Liu H., Guo X., Gooneratne R., Lai R., Zeng C., Zhan F., Wang W. (2016). The gut microbiome and degradation enzyme activity of wild freshwater fishes influenced by their trophic levels. Sci. Rep..

[8] Wu S., Ren Y., Peng C., Hao Y., Xiong F., Wang G., Li W., Zou H., Angert E.R. (2015). Metatranscriptomic discovery of plant biomass-degrading capacity from grass carp intestinal microbiomes. FEMS Microbiol. Ecol..

[9] Dearing M.D., Kohl K.D. (2017). Beyond fermentation: Other important services provided to endothermic herbivores by their gut microbiota. Integr. Comp. Biol..

[10] Wilson A.S., Koller K.R., Ramaboli M.C., Nesengani L.T., Ocvirk S., Chen C., Flanagan C.A., Sapp F.R., Merritt Z.T., Bhatti F. (2020). Diet and the human gut microbiome: An international review. Digest. Dis. Sci..

[11] Agboola J.O., Chikwati E.M., Hansen J.Ø., Kortner T.M., Mydland L.T., Krogdahl Å., Djordjevic B., Schrama J.W., Øverland M. (2022). A meta-analysis to determine factors associated with the severity of enteritis in Atlantic salmon (*Salmo salar*) fed soybean meal-based diets. Aquaculture.

[12] Zhang W., Tan B., Deng J., Haitao Z. (2021). Multiomics analysis of soybean meal induced marine fish enteritis in juvenile pearl gentian grouper, *Epinephelus fuscoguttatus*♀ × *Epinephelus lanceolatus*♂. Sci. Rep..

[13] Infante-Villamil S., Huerlimann R., Jerry D.R. (2021). Microbiome diversity and dysbiosis in aquaculture. Rev. Aquac..

[14] Coyte K.Z., Schluter J., Foster K.R. (2015). The ecology of the microbiome: Networks, competition, and stability. Science.

[15] Montoya J.M., Pimm S.L., Solé R.V. (2006). Ecological networks and their fragility. Nature.

[16] Yang G., Jian S.Q., Cao H., Wen C., Hu B., Peng M., Peng L., Yuan J., Liang L. (2019). Changes in microbiota along the intestine of grass carp (*Ctenopharyngodon idella*): Community, interspecific interactions, and functions. Aquaculture.

[17] Zhu B., Wang X., Li L. (2010). Human gut microbiome: The second genome of human body. Protein Cell.

[18] Aron-Wisnewsky J., Warmbrunn M.V., Nieuwdorp M., Clément K. (2021). Metabolism and metabolic disorders and the microbiome: The intestinal microbiota associated with obesity, lipid metabolism, and metabolic health—Pathophysiology and therapeutic strategies. Gastroenterology.

[19] Portincasa P., Bonfrate L., Vacca M., De Angelis M., Farella I., Lanza E., Khalil M., Wang D.Q.-H., Sperandio M., Di Ciaula A. (2022). Gut Microbiota and Short Chain Fatty Acids: Implications in Glucose Homeostasis. Int. J. Mol. Sci..

[20] Lin R., Liu W., Piao M., Zhu H. (2017). A review of the relationship between the gut microbiota and amino acid metabolism. Amino Acids.

[21] Nieuwdorp M., Gilijamse P.W., Pai N., Kaplan L.M. (2014). Role of the microbiome in energy regulation and metabolism. Gastroenterology.

[22] Tran N.T., Zhang J., Xiong F., Wang G.-T., Li W.-X., Wu S.-G. (2018). Altered gut microbiota associated with intestinal disease in grass carp (*Ctenopharyngodon idellus*). World J. Microb. Biot..

[23] Sun B.-Y., Yang H.-X., He W., Tian D.-Y., Kou H.-Y., Wu K., Yang C.-G., Cheng Z.-Q., Song X.-H. (2021). A grass carp model with an antibiotic-disrupted intestinal microbiota. Aquaculture.

[24] Zhang J., Xiong F., Wang G.T., Li W.X., Li M., Zou H., Wu S.G. (2017). The influence of diet on the grass carp intestinal microbiota and bile acids. Aquac. Res..

[25] Wu S., Wang G., Angert E.R., Wang W., Li W., Zou H. (2012). Composition, diversity, and origin of the bacterial community in grass carp intestine. PLoS ONE.

[26] Tran N.T., Wang G.T., Wu S.G. (2017). A review of intestinal microbes in grass carp *Ctenopharyngodon idellus* (Valenciennes). Aquac. Res..

[27] Yang G., Xu Z., Tian X., Dong S., Peng M. (2015). Intestinal microbiota and immune related genes in sea cucumber (*Apostichopus japonicus*) response to dietary β-glucan supplementation. Biochem. Biophys. Res. Commun..

[28] Rognes T., Flouri T., Nichols B., Quince C., Mahé F. (2016). VSEARCH: A versatile open source tool for metagenomics. PeerJ.

[29] Carrión V.J., Perez-Jaramillo J., Cordovez V., Tracanna V., De Hollander M., Ruiz-Buck D., Mendes L.W., van Ijcken W.F., Gomez-Exposito R., Elsayed S.S. (2019). Pathogen-induced activation of disease-suppressive functions in the endophytic root microbiome. Science.

[30] Anderson M.J. (2015). Permutational multivariate analysis of variance. Dep. Stat. Univ. Auckl. Auckl..

[31] Deng Y., Jiang Y.-H., Yang Y., He Z., Luo F., Zhou J. (2012). Molecular ecological network analyses. BMC Bioinf..

[32] Douglas G.M., Maffei V.J., Zaneveld J.R., Yurgel S.N., Brown J.R., Taylor C.M., Huttenhower C., Langille M.G. (2020). PICRUSt2 for prediction of metagenome functions. Nat. Biotechnol..

[33] Clarke K.R. (1993). Non-parametric multivariate analyses of changes in community structure. Aust. Ecol..

[34] de Vos W.M., Tilg H., Van Hul M., Cani P.D. (2022). Gut microbiome and health: Mechanistic insights. Gut.

[35] Mallott E.K., Amato K.R. (2021). Host specificity of the gut microbiome. Nat. Rev. Microbiol..

[36] David L.A., Maurice C.F., Carmody R.N., Gootenberg D.B., Button J.E., Wolfe B.E., Ling A.V., Devlin A.S., Varma Y., Fischbach M.A. (2014). Diet rapidly and reproducibly alters the human gut microbiome. Nature.

[37] Feng W., Zhang J., Jakovlić I., Xiong F., Wu S., Zou H., Li W., Li M., Wang G. (2019). Gut segments outweigh the diet in shaping the intestinal microbiota composition in grass carp *Ctenopharyngodon idellus*. AMB Express.

[38] Fečkaninová A., Koščová J., Mudroňová D., Popelka P., Toropilova J. (2017). The use of probiotic bacteria against Aeromonas infections in salmonid aquaculture. Aquaculture.

[39] Igbinosa I.H., Beshiru A., Odjadjare E.E., Ateba C.N., Igbinosa E.O. (2017). Pathogenic potentials of *Aeromonas* species isolated from aquaculture and abattoir environments. Microb. Pathogen..

[40] Song X., Zhao J., Bo Y., Liu Z., Wu K., Gong C. (2014). *Aeromonas hydrophila* induces intestinal inflammation in grass carp (*Ctenopharyngodon idella*): An experimental model. Aquaculture.

[41] Song X., Hu X., Sun B., Bo Y., Wu K., Xiao L., Gong C. (2017). A transcriptome analysis focusing on inflammation-related genes of grass carp intestines following infection with *Aeromonas hydrophila*. Sci. Rep..

[42] Dong Y., Yang Y., Liu J., Awan F., Lu C., Liu Y. (2018). Inhibition of *Aeromonas hydrophila*-induced intestinal inflammation and mucosal barrier function damage in crucian carp by oral administration of *Lactococcus lactis*. Fish Shellfish Immunol..

[43] Gewaily M.S., Shukry M., Abdel-Kader M.F., Alkafafy M., Farrag F.A., Moustafa E.M., Doan H.V., Abd-Elghany M.F., Abdelhamid A.F., Eltanahy A. (2021). Dietary *Lactobacillus plantarum* relieves Nile tilapia (*Oreochromis niloticus*) juvenile from oxidative stress, immunosuppression, and inflammation induced by deltamethrin and *Aeromonas hydrophila*. Front. Mar. Sci..

[44] Zhou J., Deng Y., Luo F., He Z., Yang Y. (2011). Phylogenetic molecular ecological network of soil microbial communities in response to elevated CO_2_. mBio.

[45] Pande S., Kaftan F., Lang S., Svatoš A., Germerodt S., Kost C. (2016). Privatization of cooperative benefits stabilizes mutualistic cross-feeding interactions in spatially structured environments. ISME J..

[46] Ren X., Murray R.M. Cooperation enhances robustness of coexistence in spatially structured consortia. Proceedings of the 2019 18th European Control Conference (ECC).

[47] Olesen J.M., Bascompte J., Dupont Y.L., Jordano P. (2007). The modularity of pollination networks. Proc. Natl. Acad. Sci. USA.

[48] Hill D., Sugrue I., Arendt E., Hill C., Stanton C., Ross R.P. (2017). Recent advances in microbial fermentation for dairy and health. F1000Research.

[49] Tremaroli V., Bäckhed F. (2012). Functional interactions between the gut microbiota and host metabolism. Nature.

[50] Shefat S.H.T., Karim M.A. (2018). Nutritional diseases of fish in aquaculture and their management: A review. Acta Sci. Pharm. Sci..

[51] Yao T., Gu X., Liang X., Fall F.N., Cao A., Zhang S., Guan Y., Sun B., Xue M. (2021). Tolerance assessment of dietary bile acids in common carp (*Cyprinus carpio* L.) fed a high plant protein diet. Aquaculture.

[52] Million M., Lagier J.-C., Yahav D., Paul M. (2013). Gut bacterial microbiota and obesity. Clin. Microbiol. Infect..

[53] Aoun A., Darwish F., Hamod N. (2020). The influence of the gut microbiome on obesity in adults and the role of probiotics, prebiotics, and synbiotics for weight loss. Prev. Nutr. Food Sci..

[54] Ni J., Yan Q., Yu Y., Zhang T. (2014). Factors influencing the grass carp gut microbiome and its effect on metabolism. FEMS Microbiol. Ecol..

[55] Leo V.V., Asem D., Singh B.P., Singh J.S., Singh D.P. (2018). Actinobacteria: A highly potent source for holocellulose degrading enzymes. Actinobacteria, New and Future Developments in Microbial Biotechnology and Bioengineering.

[56] Smid E., Lacroix C. (2013). Microbe–microbe interactions in mixed culture food fermentations. Curr. Opin. Biotechnol..

[57] Beltrán J., Esteban M. (2022). Nature-identical compounds as feed additives in aquaculture. Fish Shellfish Immunol..

[58] Vargas-Albores F., Martínez-Córdova L., Hernández-Mendoza A., Cicala F., Lago-Lestón A., Martínez-Porchas M. (2021). Therapeutic modulation of fish gut microbiota, a feasible strategy for aquaculture?. Aquaculture.

