# Supplementary material for: Response of Intestinal Microbiota to the Variation in Diets in Grass Carp (Ctenopharyngodon idella)
Source: Metabolites. 2022 Nov 15;12(11):1115. doi: 10.3390/metabo12111115 (PMC9698803; doi:10.3390/metabo12111115)
Supplement: Supplementary file 1 [file metabolites-12-01115-s001.zip › metabolites-2010536-Supplementary.pdf]

Supplemental data

Table S1 Relative abundance of different bacterial phyla in grass carp.

Percentage (%)	GF	CF
Firmicutes	20.49	61.59
Proteobacteria	56.59	25.42
Actinobacteria	10.77	2.97
Fusobacteria	0.24	5.62
Chloroflexi	3.93	1.02
Acidobacteria	2.19	0.14
Thaumarchaeota	1.19	0.04
Other	4.61	3.22

Table S2 Relative abundance of different bacterial classes in grass carp.

Percentage (%)	GF	CF
Bacilli	16.11	55.58
Alphaproteobacteria	43.84	16.63
Gammaproteobacteria	9.51	7.51
Actinobacteria	10.77	2.97
Clostridia	3.70	5.24
Fusobacteriia	0.24	5.62
Betaproteobacteria	1.58	0.65
Deltaproteobacteria	1.40	0.36
Caldilineae	1.02	0.25
Other	11.84	5.19
